# Comparative study of imaging and histology of sacroiliac joint in normal rats based on IVIM-DWI and DCE-MRI

**DOI:** 10.1186/s12891-020-03481-1

**Published:** 2020-07-20

**Authors:** Jian Qin, Qianqian Yao, Xubo Ge, Jianzhong Zhu, Zhaoliang Yin, Xiaoqian Li, Changqin Li

**Affiliations:** 1 Department of Radiology, The Second Affiliated Hospital of Shandong First Medical University, No. 366 Taishan Street, Taian City, 271000 Shandong China; 2 Department of Radiology, The Fourth People’s Hospital of Taian, Taian, 271000 Shandong China

**Keywords:** Sacroiliac joint, Intravoxel incoherent motion, Dynamic contrast-enhanced MRI

## Abstract

**Background:**

Currently, few studies have described the relationship between functional MRI findings and histology of normal sacroiliac joint (SIJ). Besides, due to the difficulties in access to SIJ, authentic animal models are important in providing opportunities for quantitative parameter extraction on imaging.

**Aims:**

This study aimed at exploring the parameters of Intravoxel Incoherent Motion Diffusion-Weighted Imaging (IVIM-DWI) and Dynamic Contrast-Enhanced Magnetic Resonance Imaging (DCE-MRI) and comparing them with the histology of the SIJ in normal rats with different ages.

**Methods:**

A total of thirty 7-week-old male Wistar rats were included in the study. The parameters of IVIM-DWI and DCE-MRI in the bone marrow and the joint space of SIJ were measured at 8, 13, 18, 23, 28, and 33 weeks. The histological analysis of the SIJ was examined using light microscopy. One-way ANOVA was used for statistical analysis.

**Results:**

The D values in the sacral and iliac bone marrow of normal rats decreased with an increase in age. One-way ANOVA analysis indicated a significant difference in D values in different age groups (*P*<0.005). The normal values of D*, f, Fenh (%), Senh (%/s) in the sacral bone marrow, the iliac bone marrow, and the joint space in SIJ of normal rats were obtained. The results showed that in the six groups of rats of different ages, the histology of the SIJ surface was smooth and clear, the cartilage cells were intact, and no thickening or pannus formation was observed.

**Conclusions:**

This study obtained the IVIM-DWI and DCE-MRI parameters of the sacral and iliac bone marrow and the synovial area of the joint space in normal rats. The parameters in normal rats can be used in future research to compare to similar parameters in animal models or patients with SIJ diseases. This study serves as a guide for future research in SIJ diseases.

## Background

The SIJ is an auricular shaped joint known to transfer weight and forces between the lumbar spine and lower limbs. The auricular joint surface connects the sacral and the iliac bones and it is surrounded by strong ligaments [1, 2]. Considering its functional ability, the SIJ has limited motion capabilities. Therefore, the main function of SIJ is to maintain body stability. Sacroiliitis is considered to be an important criterion for the diagnosis and classification of ankylosing spondylitis [3]. To understand the pathologies arising in the SIJ, it is very important to understand the normal anatomy, histology, and imaging characteristics of the normal SIJ. Previous studies have focused on some parts of the descriptive anatomy, diagnosis, and treatment of SIJ pathologies [4, 5]. Other studies have also emerged on the biomechanics of the SIJ, in terms of the structural relationships of the joint with surrounding tissues [6, 7]. In recent years, modern imaging technologies have been widely studied for early diagnosis of arthritis [8]. However, there is a lack of comparative studies on imaging analysis of the normal SIJ. Some scholars have performed pathological and related MR examinations of the SIJ using the male corpse [9]. However, these studies do not reflect the physiological condition of a normal and abnormal SIJ in the living state and cannot be used to extract quantitative imaging parameters. Therefore, few studies have described the relationship between functional MRI findings and histology of normal SIJ. Besides, due to the difficulties in access to SIJ, authentic animal models are important in providing opportunities for quantitative parameter extraction on imaging.

In this study, we hypothesized that there is a relationship between the IVIM-DWI and DCE-MRI parameters and the histological findings of normal SIJ. Therefore, in this study, MR parameters of IVIM-DWI and DCE-MRI were measured in rats of different age groups and compared with histological findings.

## Methods

### Research objects

Thirty male Wistar rats were obtained from Experimental Animal breeding Co., Ltd. (China, Jinan). The rats were 7 weeks old and weighed 170-200 g. All rats were acclimatized in a Specific Pathogen Free (SPF) environment for 1 week before the experiment. At 8, 13, 18, 23, 28, and 33 weeks, 5 rats were randomly selected for MRI examination under anesthesia (3 ml of Urethane intraperitoneal injection, Shanghai Shanpu Chemical Co., Ltd), then euthanized and sent for histological analysis. A 0.2 g/mL solution of Urethane was prepared using saline (total of 10 ml/kg) with intraperitoneal injection. The study was approved by the Institutional Animal Care and Use Committee of Shandong First Medical University and was performed in accordance with the National Institutes of Health guidelines for the use of laboratory animals.

### MR imaging techniques

All MR imaging was performed using a 3.0-T MR (GE discovery MR750) and utilizing a matched eight-channel animal coil (Wankang Medical Technology Co., Ltd., China). Four standard MR imaging sequences were performed. (A) Axial T2 fat-saturated (FS) images [echo time (TE)/repetition time (TR), 96.1 ms/3000 ms; echo train length,16]; (B) Coronal T1 FS fast spin-echo (FSE) (TE/TR, 13.5 ms/500 ms; echo train length, 3); (C) IVIM-DWI: repetition time/echo time (TR/TE) 4000/66.8 ms, slice thickness 3.0 mm, matrix 64 × 64,FOV 140 mm × 112 mm, spatial resolution 3.8mm^2^, a total of 12 b-values were used: 10, 20, 30, 50, 80, 100, 200, 300, 600, 800, 1000, 1500s/mm^2^. (D) DCE-MRI: fat-saturated contrast-enhanced T1 images with the liver acquisition with volume acceleration (LAVA) sequence, repetition time/echo time (TR/TE) 5.6/1.9 ms, slice thickness 2.0 mm, matrix 128 × 128, FOV 200 mm × 160 mm. A total of 80 phases were acquired, with a spatial resolution of 2.0 mm2. 1 ml of gadopentetate dimeglumine contrast agent (BeiLu Pharmaceutical Co., Ltd., Beijing, China) was administered intravenously (tail vein) at a rate of 0.1 ml/s, followed by a 2 ml saline (0.9%, Shandong Qidu Pharmaceutical Co., Ltd) flush by hand. The concentration of gadopentetate dimeglumine was 0.5 g/ml. After the acquisition of seven baseline dynamic scans, 960 images were collected in total with 80 phases for approximately 5 min of scanning; the function tool software of GE MR Advantage Workstation 4.6 was used to perform the measurements of DCE-MRI and IVIM-DWI.

### Image analysis

#### Analysis of IVIM parameters

The DWI signal follows the biexponential model to calculate the signal attenuation IVIM, as [10]:

$$ \mathrm{S}\left(\mathrm{b}\right)/{\mathrm{S}}_0=\left(1\hbox{-} \mathrm{f}\right)\exp \left(\hbox{-} \mathrm{b}\mathrm{D}\right)+\mathrm{f}\ \exp \left[\hbox{-} \mathrm{b}\times \left(\mathrm{D}\ast +\mathrm{Dblood}\right)\right] $$

Where Sb is the signal intensity in the pixel with diffusion gradient b, S_0_ is the signal intensity without diffusion gradient, D (× 10^− 4^ mm^2^/s) is the water diffusion coefficient in the tissue, f is the perfusion fraction related to microcirculation (flowing blood fraction) and D* (× 10^− 4^ mm^2^/s) is the pseudo-diffusion coefficient which represents perfusion-related diffusion.

#### Analysis of DCE-MRI parameters

For DCE-MRI parameter analysis, all data were quantitatively analyzed using image processing software. This was calculated by manually drawing different regions of interest (ROIs), to obtain the time-intensity curves (TICs).

$$ {\displaystyle \begin{array}{l}\mathrm{Enhancement}\ \mathrm{factor}\ \mathrm{Fenh}\ \left(\%\right)=\left({\mathrm{SI}}_{\mathrm{max}}-{\mathrm{SI}}_0\right)\times 100\%/{\mathrm{SI}}_0;\\ {}\mathrm{Enhancement}\ \mathrm{slope}\ \mathrm{Senh}\ \left(\%/\mathrm{s}\right)=\left({\mathrm{SI}}_{\mathrm{max}}\hbox{-} {\mathrm{SI}}_0\right)\ 100/\left({\mathrm{SI}}_0\times {\mathrm{T}}_{\mathrm{max}}\right);\end{array}} $$

where SI_0_ (baseline signal intensity before contrast injection) approaches the SI_max_ (maximum signal intensity) exponentially in time. SI_max_ is determined as the maximum SI during the DCE-MRI examination and T_max_ is the time point of SI_max_.

#### Data analysis

**Fig. 1:**
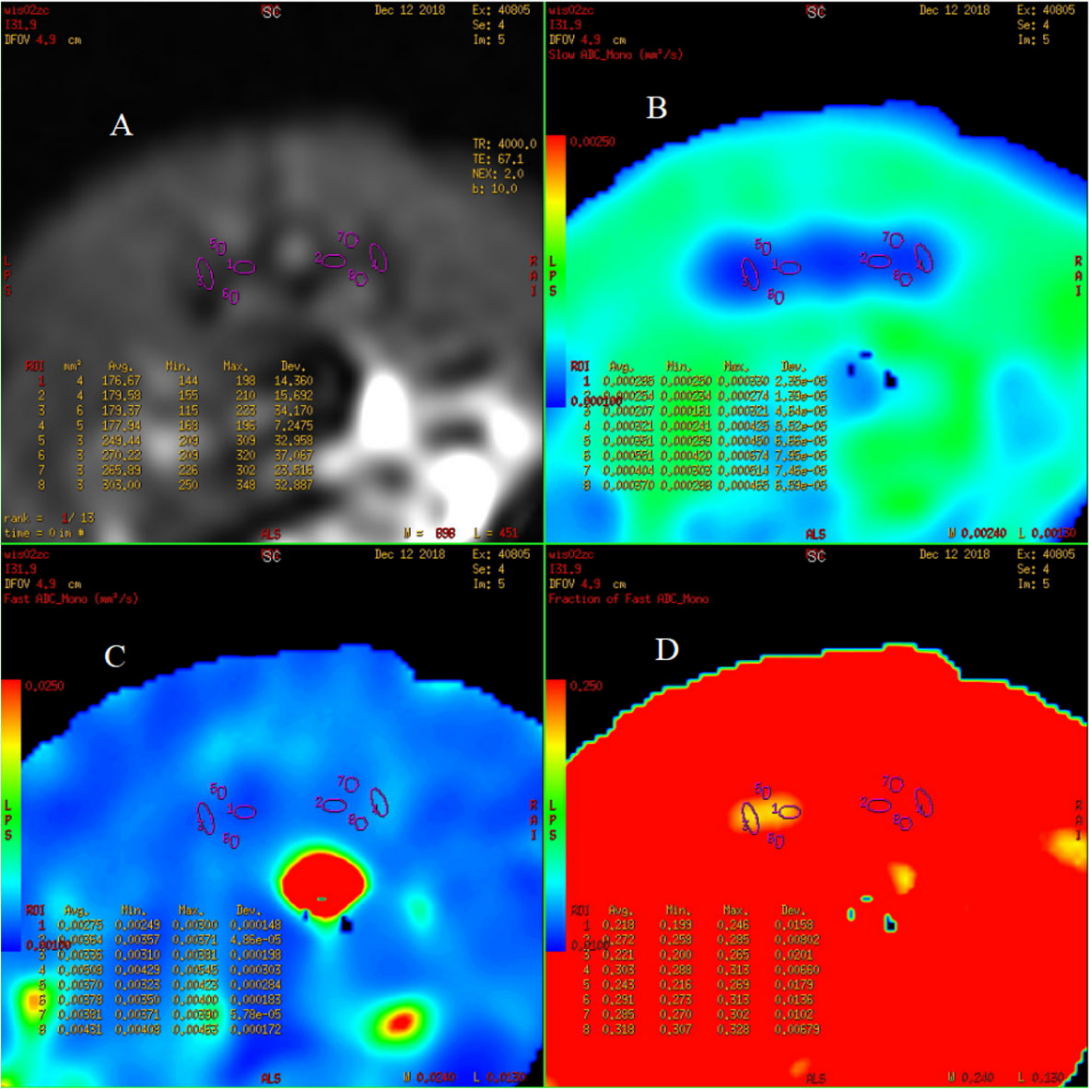
IVIM-DWI images of the SIJ in normal rats, (**a**-**d**) Axial T2-weighted FS image, D, D*, f

**Fig. 2:**
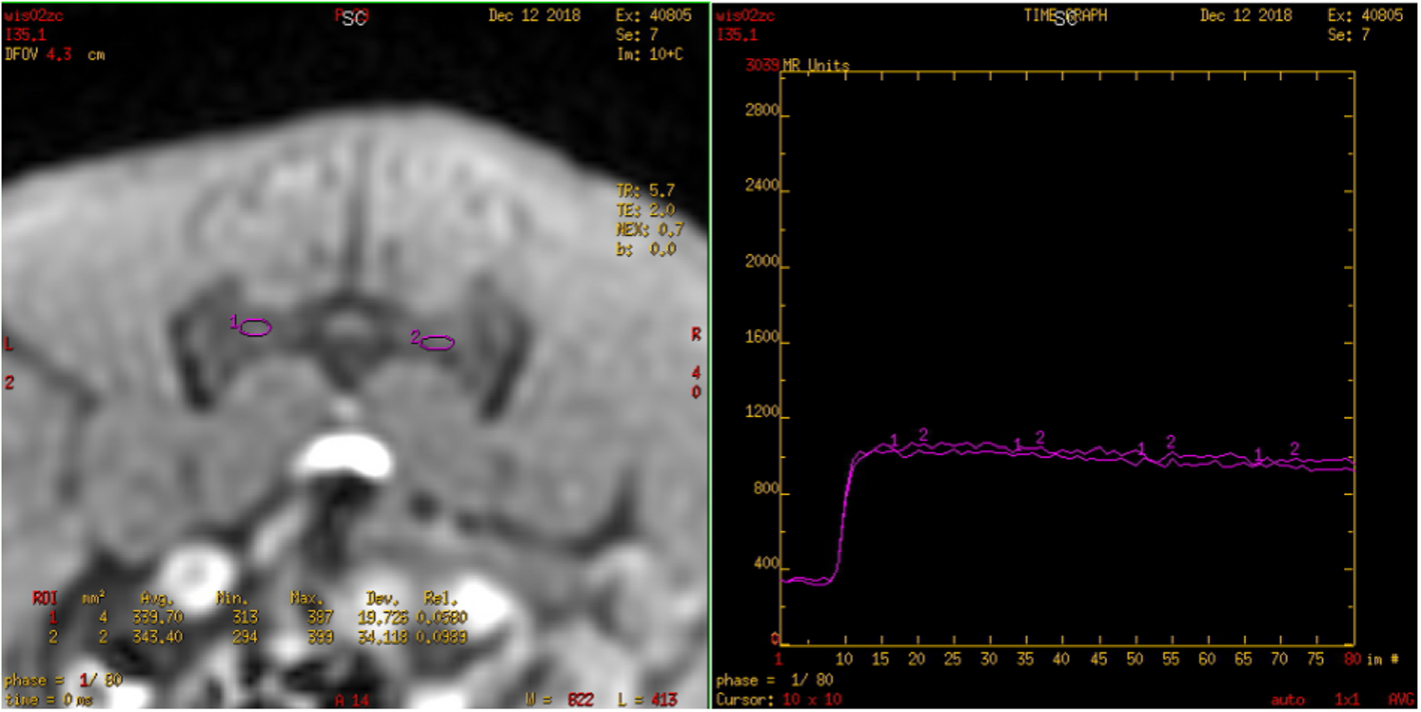
The TIC of the bilateral sacral bone marrow of the 28-week rat is a type I curve (rapid rise/steep slope and slow drop type)

**Fig. 3:**
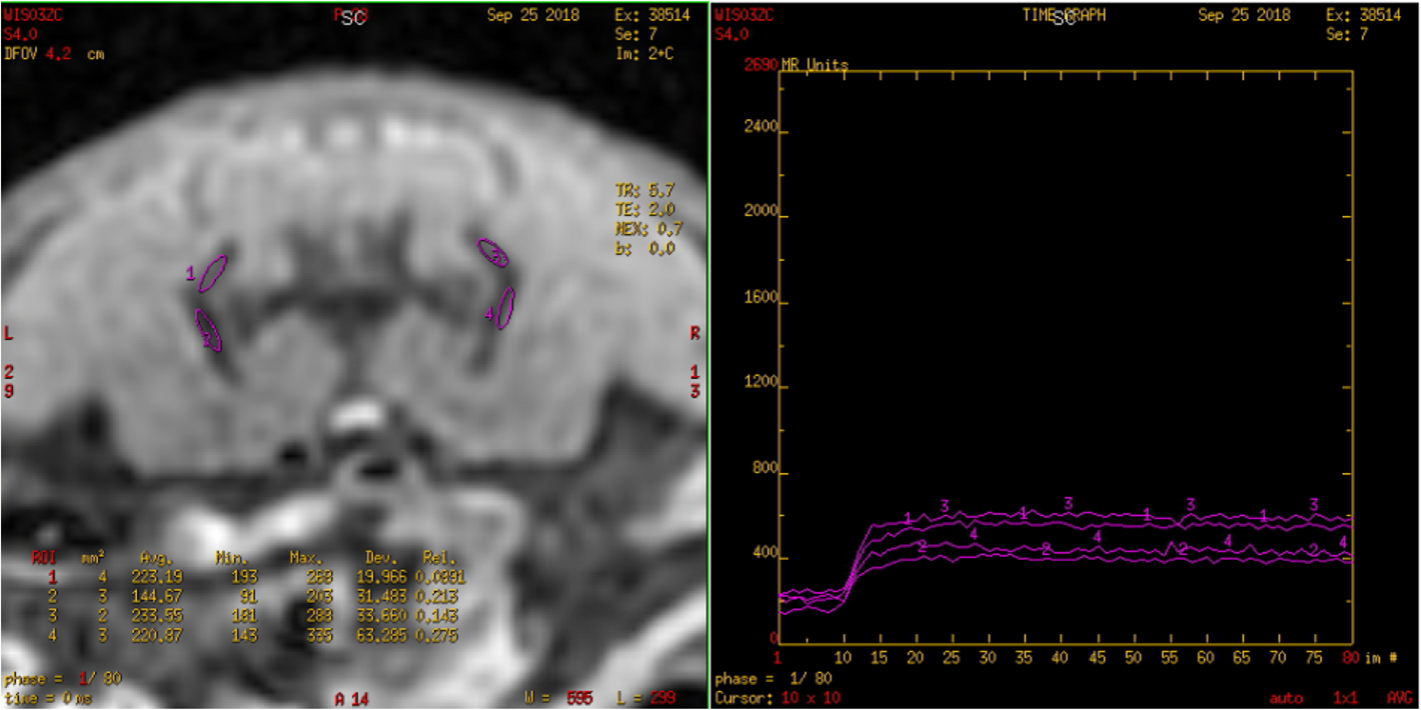
The TIC of the bilateral iliac bone marrow in the 23-week rat is a type II curve (platform/plateau type)

The original images were processed using the Advantage Workstation (ADW 4.6 version, GE, US) and post-processed by Functool workstation. Two observers with 15 years and 20 years of experience in MRI were blinded to the information and they individually measured the resulting parameter maps. Any arising disagreements of both radiologists were resolved by consensus. All data were measured 3 times and the average is taken to reduce bias caused by measurement error. On the IVIM-DWI and DCE-MRI series, an ovoid region of interest (ROI) was placed within the bilateral sacral or iliac bone marrow. The ROI of the joint space was placed at the lower third of the cartilaginous portion of the SIJ [11] and all ROIs were 2-4 mm^2^. The bilateral average values were obtained and care was taken to avoid the cortex, venous plexus, ligaments, or any imaging-related artifacts (Figs. 1, 2, 3).

### Histological assessment

After MR examination, the rats were weighed and each rat was euthanized with 1% pentobarbital (Sigma company) 100 mg/kg intraperitoneal injection. When the heartbeats were not detected for 5 min, SIJ samples were removed, fixed in 10% formalin (200 ml per sample) for 1-2 days, acid-decalcified with 10% methanoic acid for 1 week, embedded in paraffin, cut after dehydration in graded ethanol (75% ethanol 15 s, 85% ethanol 10s, 95% ethanol 10s, absolute ethanol 1 min, absolute ethanol 1 min,) and stained with hematoxylin (8 min) and eosin (2 min). The histological changes of the SIJ were observed under a microscope (MODEL BX53F, OLYMPUS, Tokyo, Japan).

### Statistical analysis

SPSS Statistics version 19.0 was used to perform statistical analysis. The test for homogeneity of variances was performed using Levene’s tests. All the parameter values were compared by one-way ANOVA. The measured parameters were expressed as means ± standard deviation (SD). *P* values < 0.05 were considered statistically significant.

## Results

### Performance of Wistar rats

Over the entire study period, there was no statistically significant difference in appearance (including paw, hair, tail, spine, etc.) among the five groups of male Wistar rats of different ages.

### Results of the various parameters of the SIJ in normal rats from the IVIM-DWI sequence

The values of D (× 10^− 4^ mm^2^/s) in the sacrum and the iliac bone marrow of normal rats in different age groups decreased with an increase in age. One-way ANOVA analysis indicated a significant difference in D values among different age groups (*P* = 0.000) (Table 1-2). Besides, there was no statistically significant difference among the D, D*and f values of the joint space in SIJ in different age groups (*P* > 0.05). The normal values of corresponding parameters are shown in Table 3. IVIM-DWI images of the SIJ in normal rats are shown in Fig. 1.

**Table 1:** IVIM parameters among different age groups in the bone marrow of the sacrum

–	D (× 10^− 4^ mm^2^/s)	D* (× 10^− 4^ mm^2^/s)	f(%)
8 weeks	4.84 ± 0.43	28.99 ± 4.80	24.45 ± 4.84
13 weeks	4.63 ± 0.40	27.37 ± 3.87	26.83 ± 3.97
18 weeks	4.76 ± 0.61	27.76 ± 3.18	25.54 ± 4.87
23 weeks	4.34 ± 0.63	26.50 ± 2.89	24.81 ± 2.89
28 weeks	3.89 ± 0.34	26.88 ± 4.19	24.63 ± 3.26
33 weeks	3.67 ± 0.37	25.66 ± 2.80	24.36 ± 3.54
*F*	26.673	1.581	1.152
*P*	0.000*	0.157	0.336

The values of D, D*, and f are presented as means ± standard deviation in the bone marrow of the sacrum. * indicates a significant difference

**Table 2:** IVIM parameters among different age groups in the bone marrow of the iliac bone

–	D(×10^−4^ mm^2^/s)	D* (×10^− 4^ mm^2^/s)	f(%)
8 weeks	5.23 ± 0.54	30.41 ± 4.80	27.00 ± 3.93
13 weeks	4.84 ± 0.61	29.84 ± 4.52	27.35 ± 3.64
18 weeks	4.90 ± 0.72	30.11 ± 4.96	26.71 ± 3.01
23 weeks	4.73 ± 0.70	29.15 ± 3.97	25.91 ± 2.99
28 weeks	3.94 ± 0.51	29.31 ± 6.16	27.61 ± 3.33
33 weeks	3.83 ± 0.45	29.08 ± 5.77	27.48 ± 3.04
*F*	19.290	0.189	0.446
*P*	0.000*	0.980	0.847

The values of D, D*, and f are presented as means ± standard deviation in the bone marrow of the iliac bone. * indicates a significant difference

**Table 3:** Normal values of IVIM-DWI parameters in the sacral and iliac bone marrow and the joint space in the SIJ of normal rats

	sacral bone marrow	iliac bone marrow	the joint space
D(×10^−4^ mm^2^/s)	–	–	6.28 ± 0.28
D*(×10^−4^ mm^2^/s)	27.31 ± 3.90	29.62 ± 5.14	61.85 ± 14.93
f(%)	25.21 ± 3.79	26.98 ± 3.38	19.85 ± 3.82

The values of D, D*, and f are presented as means ± standard deviation

### Results of the various parameters of SIJ in normal rats from DCE-MRI sequence

The values of Fenh (%) and Senh (%/s) in the sacral and iliac bone marrow and the joint space in the SIJ were not significantly different from an increase in age (*P* > 0.05). The normal values of corresponding parameters are shown in Table 4. Based on observation and analysis of the dynamic enhancement curve, the sacral bone marrow showed a type I curve (rapid rise and slow drop, Fig. 2); the iliac bone marrow and the joint space in the SIJ of normal rats showed a type II curve (platform, Fig. 3).

**Table 4:** Normal values of DCE-MRI parameters in the sacral and iliac bone marrow and the joint space in the SIJ of normal rats

	sacral bone marrow	iliac bone marrow	the joint space
Fenh (%)	205.26 ± 21.04	202.87 ± 40.56	194.19 ± 24.41
Senh(%/s)	3.93 ± 1.22	3.17 ± 1.34	2.51 ± 0.41

The values of Fenh (%), Senh (%/s) are presented as means ± standard deviation

### Histological changes

**Fig. 4:**
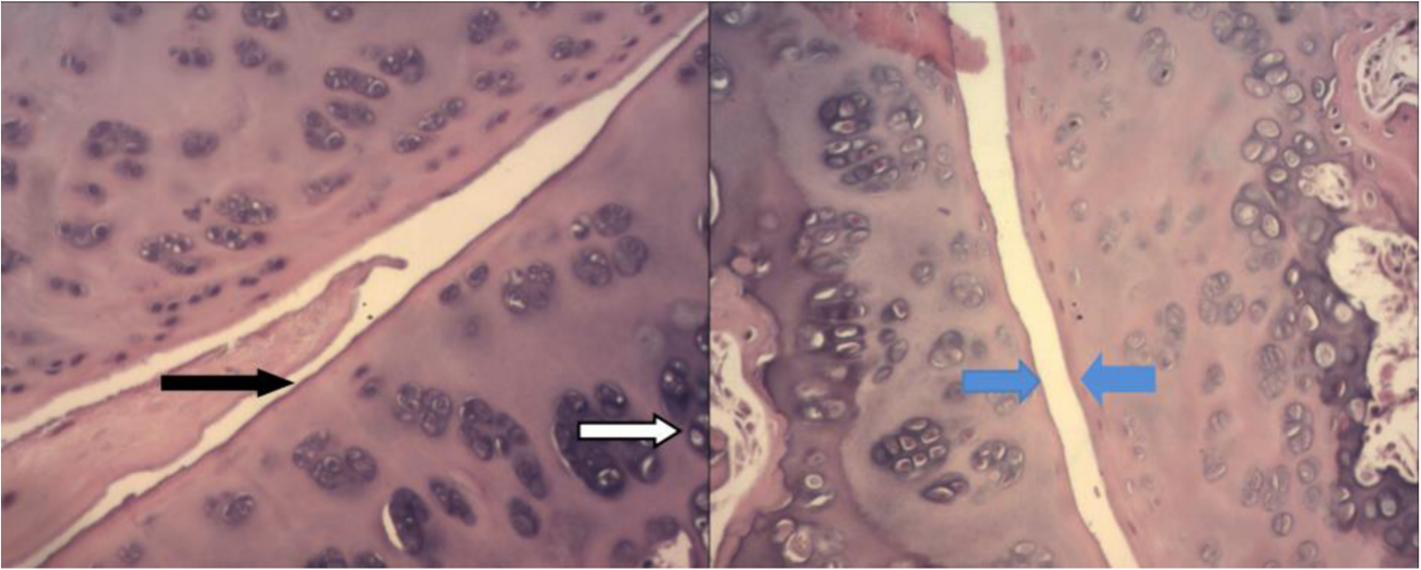
Clearly showing the synovium (black arrow), chondrocytes (white arrow), sacroiliac surface (blue arrow). The magnification is 200 ×

The six groups of rats with different ages had a smooth and clear SIJ surface, the cartilage cells were intact, the synovium was normal, and no thickening or pannus formation was observed (Fig. 4).

## Discussion

The SIJ participates in stabilizing the body movements and helps to maintain balance. The complex anatomical structure and deep position of the SIJ make it difficult to conduct general examinations. Therefore, the diagnostic evaluation of the SIJ is mainly based on medical imaging modalities. Plain X-ray examination can only show the general shape of the SIJ, and the value of X-ray in clinical diagnosis, treatment, and follow-up is insufficient. CT clearly shows the osteoarticular surfaces and subarticular trabecular bone, however, the display of articular cartilage and bone marrow changes is insufficient [12]. MRI can clearly show the bone marrow cavity, the articular cartilage, and the surrounding soft tissue. Bone Marrow Edema (BME) is relatively easy to depict on short tau inversion recovery (STIR) images and has a high sensitivity in the diagnosis of SpA. However, the extent of the lesion cannot be quantified [13, 14]. Therefore, the application of new MRI scanning technologies like IVIM-DWI and DCE-MRI can gradually compensate for these shortcomings.

Due to the difficulties in the histological analysis of SIJ, authentic animal models are important in providing opportunities for quantitative parameter extraction on imaging. In this study, the rat model of sacroiliac arthritis was successfully established by using bovine proteoglycan combined with complete/incomplete Freund’s adjuvant. The results showed that synovitis was an early pathological manifestation of sacroiliac arthritis, and these findings were consistent with the findings in other studies conducted on human subjects [15]. As a control group, our study lays a foundation for the pathogenesis of ankylosing spondylitis in rats and provides opportunities to investigate their histology in relationship to human disease.

The apparent diffusion coefficient (ADC) is the most commonly used metric in the mono-exponential model (MEM) of DWI, which does not consider the influence of the microcirculation of blood in capillaries, thus leading to an inaccurate description of the diffusion process [16]. Besides, two main aspects affect the measured diffusion signals in living tissues, the motion of water molecules, and the perfusion of the tissue microvasculature. In 1986, Le Bihan et al. described a new imaging technique referred to as IVIM using multi b-value DWI with a bi-exponential curve fitting [10]. This technique is sensitive not only to molecular diffusion in tissues but also to random blood flow in capillaries. The technique also provides related analytical parameters represented by diffusion-related parameters D (pure molecular diffusion) and perfusion-related parameters including D* (pseudo-diffusion coefficient) and f (perfusion fraction). Semi-quantitative DCE-MRI using a generalized kinetic model allows for estimation of tissue perfusion and capillary permeability, which involves not only exchange dynamics between extra-vascular/extra-cellular space, but also some other factors, such as the pattern of blood delivery, blood vessel density, vascular permeability, and distribution of contrast agent in lesions [17]. Numerous studies have demonstrated the efficiency of IVIM-DWI and DCE-MRI parameters in prostate cancer [18], breast cancer [19], and liver fibrosis [20]. However, very few studies have been reported on SIJ.

In this study, the results showed that the D-values of the sacral and iliac bone marrow decreased with an increase in age and the difference was found to be statistically significant (*P* = 0.000). However, D* and f values did not show statistically significant differences in the groups. The reason could have been that the main components of the bone marrow cavity of the normal sacrum and ilium are the bone trabeculae and bone marrow. The main components of bone trabeculae are minerals such as calcium salts and almost no water molecules can be found, therefore, little to no effect on MR images is expected. The bone marrow is divided into two types: the yellow and red bone marrow. The bone marrow cavity in a newborn is filled with the red bone marrow. With increasing age, part of the red bone marrow is replaced with the yellow bone marrow. The red bone marrow contains different stages of red blood cells, white blood cells, platelets, and the cellular component contains a lot of water. However, the main component of the yellow bone marrow is fat, hence, the ratio of water molecules contained in the two different types of bone marrow is different. Water content and different states of water molecules can affect the image characteristics and corresponding parameters [21]. Rats aged 8 weeks to 32 weeks old correspond to human beings aged 12 to 20 years old, and the bone marrow in the SIJ in this age has begun to transform from red bone marrow to yellow bone marrow. At the same time, the content of free water molecules in the bone marrow decreases gradually. Therefore, the D value reflecting the true water molecule diffusion also decreases with an increase in age. However, there was no lesion, no inflammatory reaction, or necrosis. Therefore, there was no significant difference in D*and f values with the increase of age.

In this study, the initial signal intensity (SI_0_), the maximum signal intensity (SI_max_), and the peak time (T_max_) were obtained using semi-quantitative analysis. The enhancement slope and factor were obtained by the signal time-intensity curve (TIC) of the region of interest in normal rats. There were 180 TIC curves of the SIJ bone marrow region (sacral, iliac) and of the synovial area of the joint space. On the sacral side, a type I curve was observed (with a rapid rise and slow drop). In the synovial area and at the iliac side, a type II curve was observed (with a rapid rise and a platform). There were no significant differences in Fenh (%) and Senh (%/s) between the sacral and iliac bone marrow with increasing age.

The results showed that in the six groups of rats of different ages, the histology of the SIJ surface was smooth and clear, the cartilage cells were intact, and no thickening or pannus formation was observed.

The use of IVIM-DWI and DCE-MRI as functional imaging modalities in normal rats to investigate the histology of the normal bone marrow and SIJ is an unmet need in the field of SIJ research. Therefore, the parameters in normal rats obtained in this study can be used in future research to compare with parameters in human patients with sacroiliac joint diseases. Differences in quantitative parameters are of great importance to understand the physiology and pathophysiology of normal versus diseased SIJs. The anatomopathological features of the normal SIJs in rats indicate the absence of sacroiliac joint diseases. Therefore, this study can guide future research in SIJ diseases.

This study was funded by the Natural Science of Shandong Provincial Foundation, China (No.ZR2017MH105), and Academic Promotion Programme of Shandong First Medical University (No.2019QL017).

## Limitations

The sample size in the study is relatively small. The correlation between diagnostic imaging and histopathology was not studied in detail. Besides, the spatial resolution of the IVIM-DWI scan was low. Furthermore, the ADC value was not calculated along with the D value to make our data more easily comparable with other studies.

## Conclusions

In conclusion, histological findings, IVIM-DWI and DCE-MRI parameters of sacrum, ilium bone marrow, and synovial area of joint space in normal rats are presented in this study. These parameters can be used in future research to compare with parameters in animal models or patients with SIJ diseases.

## Data Availability

The datasets used and/or analyzed during the current study are available from the corresponding author on reasonable request.

## Abbreviations

SIJ: Sacro-Iliac Joint
IVIM-DWI: Intravoxel Incoherent Imaging Diffusion-Weighted Imaging
DCE-MRI: Dynamic Contrast-Enhanced Magnetic resonance imaging
ROI: Region of Interest
TIC: Time-Intensity Curve
ANOVA: Analysis of Variance
